# ‘I think I’m gonna hurl’: A Narrative Review of the Causes of Nausea and Vomiting in Sport

**DOI:** 10.3390/sports7070162

**Published:** 2019-07-04

**Authors:** Patrick B. Wilson

**Affiliations:** Human Movement Sciences, Old Dominion University, Norfolk, VA 23529, USA; pbwilson@odu.edu; Tel.: +757-683-4783; Fax: +757-683-4270

**Keywords:** endurance exercise, gastrointestinal, gut, sports medicine

## Abstract

Exercise-associated gastrointestinal (GI) distress can negatively impact athletic performance and interfere with exercise training. Although there are a few universal underlying causes of GI distress, each symptom often has its own unique triggers and, therefore, its own prevention and management strategies. One of the most troubling GI symptoms an athlete can experience during training and competition is nausea/vomiting. The prevalence of nausea varies with several factors, two of the most important being exercise intensity and duration. Relatively brief, high-intensity exercise (e.g., sprinting, tempo runs) and ultra-endurance exercise are both associated with more frequent and severe nausea. The potential causes of nausea in sport are numerous and can include catecholamine secretion, hypohydration, heat stress, hyponatremia, altitude exposure, excessive fluid/food consumption, hypertonic beverage intake, pre-exercise intake of fatty- or protein-rich foods (especially in close proximity to exercise), prolonged fasting, various supplements (caffeine, sodium bicarbonate, ketones), certain drugs (antibiotics, opioids), GI infections, and competition-related anxiety. Beyond directly addressing these aforementioned causes, antiemetic drugs (e.g., ondansetron) may also be useful for alleviating nausea in some competitive situations. Given the commonness of nausea in sport and its potential impact on exercise performance, athletes and sports medicine practitioners should be aware of the origins of nausea and strategies for dealing with this troublesome gut complaint.

## 1. Introduction

Exercise- and competition-associated gastrointestinal (GI) distress is a common issue encountered by athletes across a broad range of sporting endeavors. While estimates vary with the type of sport, intensity of exercise, duration of exercise, and survey methodology, the majority of athletes, particularly endurance athletes, experience some GI issues during exercise [1,2]. For example, in a recent study of 145 endurance runners, men and women experienced at least one GI symptom on 84.0% and 78.3% of their training runs over a period of 30 days [3]. Moderate-to-severe GI symptoms (score of ≥5 on a 0–10 scale) were less common but still fairly prevalent (13.8% and 21.7% of runs for men and women, respectively). 

Although some GI symptoms (e.g., flatulence, belching) may simply be a nuisance, certain symptoms can actually impair performance [4,5]. One of the most concerning of these performance-altering GI symptoms is nausea, which can be defined as a feeling of sickness in the stomach marked by an urge to vomit. Take, for example, an investigation that found, among non-finishers of 100-mile ultra-running races, nausea/vomiting was the leading reason for dropping out [6]. If nausea leads to actual vomiting, it can also contribute to fluid losses and electrolyte imbalances in severe cases. Due to its potential impact on athletic performance and health, it’s important for athletes and sports practitioners to be educated on the causes of exercise- and competition-induced nausea and vomiting and ways to prevent it. 

To date, there have been numerous review articles on the issue of GI distress in sport and exercise e.g., [1,7,8,9]. However, these articles generally took a broad perspective when looking at GI symptoms, neglecting to focus in detail on any given symptom. Importantly, although there may be some universal underlying causes of GI distress (e.g., reduced gut blood flow), each symptom often has its own unique triggers and, therefore, its own prevention and management strategies. Given this lack of focus on individual GI symptoms, the purpose of this article is to provide a narrative review of the prevalence, pathophysiology, and management strategies for exercise- and competition-associated nausea and vomiting. 

## 2. Prevalence of Nausea in Sport

Variations in the prevalence of nausea depend to a large extent on the duration and intensity of exercise. In a survey of 44 marathon participants, 11% reported nausea during 26.2-mile races, which was the same prevalence observed during 25-km races [10]. In a larger survey of 707 marathoners, roughly 12% reported at least occasionally feeling nauseated during or after self-perceived hard training runs and races [11]. In contrast, just 1.8% of the runners reported nausea during easy runs. Actual vomiting was quite rare, with 0.3% and 1.8% of runners reporting it as a symptom during easy and hard runs, respectively [11]. 

Although the occurrence of nausea is usually relatively low in races that last up to a few hours, it becomes more prevalent during ultra-endurance exercise. In a study of 70.3-mile triathlon participants (mean finish time 5–6 h), 21% of participants reported some level of nausea during the bicycle leg, which increased to 30% by the run leg [12]. Ultra-running races may make athletes particularly prone to developing nausea, as one investigation found that 60% of surveyed athletes from the 2014 Western States Endurance Run (161-km) reported nausea at some point during the race, and 25% reported vomiting at some juncture [13]. 

Information on the occurrence of nausea and vomiting in intermittent team sports is sparse. Studies have found that general GI discomfort increases during intermittent team-sport exercise [14,15], but most investigations haven’t specifically asked about nausea and vomiting. One of these studies that did inquire about nausea had 20 Brazilian junior soccer players partake in a 90-min match, with only one subject reporting nausea [16]. An experimental study involving 10 male basketball players found that mean nausea severity increased during a basketball game simulation test after eating carbohydrate and carbohydrate-protein meals 90 min before exercise, but the actual incidence among the players wasn’t reported [17]. Overall, there is a large gap in the literature on the incidence of nausea and vomiting in intermittent team-based sports like basketball, soccer, hockey, and rugby. 

Similar to the situation with team-based sports, there is a lack of literature on the prevalence of nausea and vomiting in sports that involve high-intensity anaerobic and sprinting exercise. Small experimental studies document that nausea is fairly common after sprinting exercise [18,19], but to date, large surveys of athletes from these sports have not been conducted to examine the frequency of nausea in the context of real-life training or competition. 

## 3. Pathophysiology of Nausea in Sport

Nausea has numerous potential triggers that encompass nutritional, pharmacological, environmental, and psychological domains. In all cases, the perception of nausea is believed to arise from activation of the vomiting center in the medulla oblongata within the brainstem [20]. The vomiting center can be activated directly or indirectly through pathways that interact with the GI tract, chemoreceptor trigger zone, cerebral cortex and thalamus, and vestibular system [20]. If activation of the vomiting center via these pathways is sufficiently strong, efferent motor pathways that innervate the upper GI tract, lower GI tract, diaphragm, and abdominal muscles will cause retching or vomiting. The remainder of this section details the various stimuli that can activate the vomiting center and subsequently induce nausea and vomiting in and around competition. 

### 3.1. Physiological and Environmental Factors

Exercise intensity is an important factor that modifies the risk of nausea, though it’s important to keep in mind that the interaction between exercise duration and intensity also matters. For example, the previously mentioned study of 707 marathoners revealed that the occurrence of nausea was over six times greater (roughly 12% vs. 1.8%) during hard training runs and races than during easy runs [11]. The increased prevalence of nausea with high-intensity exercise likely stems, in part, from activation of the sympathetic nervous system (SNS) and release of catecholamines into the bloodstream. Notably, concentrations of epinephrine and norepinephrine in the blood rise in a dose-dependent fashion with exercise intensity [21,22], which would support the notion that exercise intensity and nausea also rise together in a somewhat dose-dependent fashion. Additional evidence supporting the idea that catecholamines contribute to nausea during and after intense exercise comes from a report of nine patients diagnosed with pheochromocytomas and paragangliomas, which are tumors that affect the adrenal glands and extra-adrenal autonomic paraganglia [23]. These tumors can cause excess secretion of catecholamines, with common symptoms being high blood pressure, anxiety, and heart palpitations. In some patients, exercise-induced nausea and vomiting is also present, and removal of the primary tumors in the aforementioned nine patients led to a cessation of exercise–induced nausea and vomiting [23]. Finally, directly administering epinephrine to people (for various medical reasons) is known to induce nausea when large dosages are used [24,25,26]. 

In addition to exercise intensity, the modality of exercise an athlete engages in may also impact the development of nausea and vomiting. Relative to cycling, running may produce more severe GI symptoms [27], which can partly be explained by the greater degree of vertical displacement to the abdomen that’s induced by running [28]. In studies of triathletes, nausea tends to be slightly more common during the run portion of races, though the fact that the run leg is held after the bicycle leg means these comparisons are confounded by exercise duration [12,29]. Experimental research directly comparing nausea between running and cycling is scarce, and the results to date have generally been equivocal [27]. Pfeiffer et al. [2] conducted surveys of athletes participating in different endurance competitions, and the overall prevalence of serious GI symptoms (defined as >4 on a 0–9 scale) was 4% in both runners and cyclists that completed races of roughly the same duration (3–4 h). Although this study didn’t report on nausea individually, the results suggest the prevalence of nausea is unlikely to be dramatically higher during endurance running than cycling when exercise duration is taken into account. 

As noted earlier, the incidence of nausea tends to be particularly high during ultra-endurance competition. Exactly why nausea is more common with extremely prolonged exercise is not entirely clear, but two putative factors involved are hypohydration and heat stress. Indeed, a number of experiments have shown nausea to be more common or severe when individuals exercise in a hypohydrated state [30,31,32]. As an athlete loses fluid stores through sweating, splanchnic blood flow can become compromised and exacerbate GI symptoms, including nausea. Notably, GI blood flow progressively declines over time during constant moderate-to-high intensity exercise [33], which could help explain the rise in nausea severity with longer exercise durations. These reductions in body water stores and GI blood flow would likely be larger in environments that are thermally challenging, and therefore contribute to nausea. In support of this idea, Snipe et al. [34] found the incidence of nausea rose from 10% to 40% among runners when they went from exercising in temperate to hot conditions (60% VO_2max_ for 2 h). 

On a mechanistic level, hypohydration may trigger nausea by stimulating the secretion of arginine vasopressin (AVP) from the pituitary gland. Concentrations of AVP in the blood rise during moderate-to-intense exercise [35], and this increase is exaggerated when exercise is carried out in a hypohydrated state [36]. Direct administration of AVP to humans has clearly been shown to evoke feelings of nausea [37,38], supporting the hypothesis that hypohydration-mediated increases in AVP play a role in the nausea that develops during prolonged exercise, especially in the heat. Beyond the role of AVP, reductions in splanchnic blood flow may contribute to nausea by inducing a state of GI permeability, which leads to endotoxin entry into the circulation and an inflammatory response in the body [39]. 

A final environmental factor that plays a role in a smaller subset of nausea cases is altitude exposure. It’s not uncommon for ultra-endurance races to be held in mountainous regions, and when these events are held at elevations of 3,000 meters or above, the risk of altitude sickness is increased [40]. The mechanisms responsible for nausea in altitude sickness may include activation of the SNS, cerebral edema, cerebral blood flow changes, and headache-induced nausea [41,42,43].

### 3.2. Diet

Nutritional intake represents one of the more modifiable types of factors known to influence the risk of nausea and vomiting during competition. On the most basic level, the volume of a meal or beverage one consumes can impact perceptions of GI discomfort and nausea through the activation of stretch and mechanoreceptors in the stomach [44]. As it relates to athletes, this helps explain the differences in GI distress observed in experiments that have fed athletes varying volumes of water and carbohydrate beverages during exercise [45,46,47]. Specifically, when fluid intakes approach 1000 mL/h during running, ratings of stomach discomfort increase by as much as two-fold in comparison to when athletes consume more modest fluid volumes. Although these studies assessed general stomach discomfort instead of nausea, it’s highly likely that the incidence of nausea was also greater given the documented overlap between perceptions of fullness and nausea [48]. In addition to these immediate effects of fluid over-consumption on nausea, sustained excess intake of fluid can provoke nausea if it leads to severe hyponatremia and cerebral edema [49,50]. Clearly, consuming large volumes of food and/or fluid immediately before or during exercise is inadvisable when an athlete wishes to minimize the risk of nausea and vomiting. 

Timing of pre-exercise nutritional intake is also a potentially important factor to consider when it comes to exercise-associated nausea and vomiting. The joint position stand on nutrition and athletic performance from the American College of Sports Medicine, Dietitians of Canada, and the Academy of Nutrition and Dietetics recommends that the timing of a pre-event meal be individualized based on the preferences, GI tolerance, and experiences of the athlete [51]. However, little specific guidance is provided in terms of volumes or amounts of food/fluid that would be ideal in relation to different timeframes prior to exercise (<60 min, 1–2 h, > 2 h, etc.). This is largely because scant research has directly compared the effects of ingesting meals at different time points in the pre-exercise period [52]. From a theoretical perspective, however, it does make sense to avoid consuming large, energy-dense meals within 1–2 h of the onset of exercise because of their delaying effects on gastric emptying [53]. 

Beyond volume, timing, and energy density, the nutrient composition of ingested foodstuffs can also impact nausea. Concentrated hypertonic carbohydrate beverages exacerbate nausea during exercise when they are consumed at relatively high rates [54,55]. To some extent, the GI disturbances from ingesting concentrated carbohydrate drinks can be mitigated by consuming products that contain a mixture of glucose and fructose instead of glucose alone [4,56,57]. This may stem, in part, from the fact that glucose inhibits gastric emptying at high concentrations in comparison to fructose and carbohydrate polymers [58]. 

Consuming large quantities of dietary fat and protein can also induce nausea through the release of gut hormones and peptides. Ingestion of all three macronutrients, but particularly protein and fat, stimulates the release of cholecystokinin (CCK) from cells in the duodenum and jejunum [59]. CCK reduces gastric motility and emptying, and particularly when dietary fat is ingested, it can contribute to feelings of fullness and nausea in the presence of gastric distention [60]. While studies examining the effects of dietary fat on CCK secretion, gastric emptying, and perceptions of nausea during exercise are lacking, some observational evidence supports the notion that ingesting fat before competition increases the risk of nausea and vomiting [61]. Non-exercise research also supports the idea that fat-rich meals can induce nausea in some settings [62,63]. Likewise, ingesting large amounts of protein (~75 g) 90 min prior to exercise can intensify nausea in comparison to carbohydrate ingestion [17]. To summarize, consuming large quantities of fat and protein within close proximity of or during exercise may trigger nausea in some athletes, although the intensity of exercise and other factors may modify these effects. Consequently, athletes should use a trial and error approach to determine their tolerance to fat and protein ingestion around the time of competition. 

It’s often recommended that athletes avoid dietary fiber in pre-competition feedings because of its capacity to slow gastric emptying, which could, in theory, increase the severity of nausea during exercise [51]. Unfortunately, direct evidence for this recommendation is lacking in athletes, though it does make logical sense to avoid large doses of fiber (>5–10 g) in meals that are consumed within 1 to 2 h of intense exercise. 

While overconsuming nutrients like fat and fiber during the pre-exercise period can cause nausea, so can prolonged periods of fasting or under-consumption of energy, at least in a subset of individuals. For example, research on individuals undergoing surgery have found longer fasting durations to correlate with more severe nausea [64], and in comparison to overnight fasting, pre-operative administration of carbohydrate drinks reduces nausea in the post-operative period [65]. While these studies weren’t completed in athletes, they do provide supportive evidence that prolonged fasting can exacerbate nausea. The effects of fasting on nausea are probably mediated through reductions in blood glucose and the stimulation of counter-regulatory hormones like glucagon and epinephrine. 

### 3.3. Dietary Supplements

Apart from the effects of day-to-day dietary choices, nutritional supplements that are intended to enhance training, performance, and/or recovery can also be a source of nausea. Two of the most scientifically supported supplements for improving physical performance (caffeine and sodium bicarbonate) both can trigger nausea under certain circumstances. Caffeine administration raises plasma catecholamine levels, particularly epinephrine [66], and at high dosages (500 mg), the side effects of caffeine (including nausea) become more pronounced [67]. Notably, a study by Bell et al. [68] reported that the combination of exercise, caffeine, and other stimulants like ephedrine (all of which stimulate catecholamine secretion) is capable of producing such severe nausea that it can interfere with a person’s ability to complete a high-intensity exercise task. Likewise, the use of pre-workout supplements that contain caffeine and other stimulants is also associated with nausea in susceptible individuals [69]. These adverse effects of caffeine may be particularly relevant in athletes that have a specific polymorphism of the adenosine A2A receptor (*ADORA2A*) gene that is associated with greater anxiety responses to caffeine ingestion [70]. Although the author is unaware of any studies directly linking the *ADORA2A* gene to nausea responses with caffeine ingestion, this is an area that should be explored in future research. 

Sodium bicarbonate is another well-documented ergogenic aid for improving high-intensity exercise performance, but it increases the severity of most GI symptoms (including nausea), preventing many athletes from using it before competition. These GI issues tend to peak 60–90 min after ingestion [71], and the symptoms can be so severe that some athletes cannot tolerate exercising, as one recent experimental trial reported seven out of 25 athletes ceased exercise testing because of GI distress [72]. Sodium citrate is another acid-buffering supplement that is sometimes claimed to have fewer GI side effects than sodium bicarbonate, but a trial published in 2016 found nausea to be reported among 100% of participants after supplementation with various dosages of sodium citrate [73]. 

An increasingly popular supplement used by athletes is exogenous ketones, which can take several forms such as ketone esters and salts [74]. Exogenous ketones do alter substrate use during exercise, suggesting they could have ergogenic properties [75], but in at least two studies to date, administration of exogenous ketones has actually resulted in impairments in performance [76,77], which can partly be explained through their propensity to increase perceptions of nausea [77]. The likelihood that ketone supplements will cause nausea depends on dosage and the form used [78], so any athlete that’s considering using these supplements should take care to trial specific products and dosages during training. Currently, there is some evidence that ester forms of ketones cause less GI distress the ketone salts [78]. 

Electrolyte supplements, especially sodium, are popular among endurance athletes. Upwards of four out of five endurance athletes report being conscious of their sodium intake during competition [79], and over 90% of participants from some ultra-running races use sodium supplements [80]. Some athletes hold the belief that sodium supplementation can prevent nausea during prolonged exercise, but sodium intakes have been reported to be similar among athletes who do and not develop nausea and vomiting during ultra-marathons [81]. While controlled studies supplementing athletes with sodium during exercise have typically neglected to collect information on GI side effects, at least one study reported several cases of nausea with the consumption of 1800 mg of sodium over two hours of exercise [82]. Thus, it seems unlikely that sodium will help mitigate the development of nausea in most athletes and may actually trigger nausea in a minority of athletes that take large dosages during exercise.

A few other supplements that are known to provoke nausea include medium-chain triglycerides [83], glycerol [84], and iron [85]. These side effects tend to be dose-dependent, can be lessened to some degree through protocol adjustments (e.g., reducing the amount, spreading doses, taking with other foods), and often dissipate with repeated ingestion over time. Of note, glycerol was previously considered a prohibited substance by the World Anti-Doping Agency (WADA) but was removed from the list in 2018. Among the other supplements discussed in this section, only ephedrine is currently prohibited by WADA. 

### 3.4. Drugs

While many over-the-counter and pharmaceutical drugs can cause nausea and vomiting, the focus of this section is on nausea-inducing drugs that are frequently used in athlete populations. Antibiotics are used by athletes to treat various infections and prophylactically prevent GI illnesses, especially those that arise from international travel [86]. Nausea is a noted side effect of antibiotics [87], although some such as rifaximin and amoxicillin may be less likely to cause queasiness [88,89]. Because there are numerous antibiotics with different mechanisms of action and pharmacokinetic properties, athletes and sports medicine practitioners should evaluate the side effect profile of individual antibiotics that are being considered for use. 

Several analgesic medications can also elicit nausea, albeit to varying degrees. Opioids are perhaps the most likely class of analgesics to cause nausea and vomiting, as these symptoms are found in about one in five to one in three users [90]. There is a lack of data on the prevalence of opioid use among sportspeople, but given the pain from injuries experienced by many athletes and the increase in opioid use among the general population, most sports medicine practitioners will encounter an athlete using these drugs at some point or another [91]. 

Nausea is often listed as a side effect of non-steroidal anti-inflammatory drug (NSAID) use, but placebo-controlled trials haven’t been consistent in terms of finding higher rates of nausea among subjects taking NSAIDs in comparison to those taking placebo [92,93,94]. Overall, the incidence of nausea while taking NSAIDs is fairly low, particularly in comparison to opioid analgesics. 

A few other notable classes of drugs that can cause nausea include some antihypertensive medications, oral contraceptives, and antidepressants (particularly selective serotonin reuptake inhibitors) [95,96]. 

### 3.5. Infectious GI Illnesses

Although estimates vary widely between studies because of differences in data acquisition and interpretation, it’s estimated that over 200 million acute GI illnesses occur each year in the United States alone [97]. Moreover, these illnesses also frequently affect travelers, including athletes, that journey to countries that have poor food and water sanitation control measures. The bulk of these illnesses are caused by bacteria, with the most common offending organism being *Escherichia coli* [98]. Low rates of traveler’s diarrhea are observed when people travel between two low-risk countries, but the rates can be as high as 60% when travelling to Latin America, Africa, and the Indian subcontinent [99]. Watery or loose stools and abdominal pain are the most common symptoms of traveler’s diarrhea, but nausea and vomiting can also occur in 30–45% of individuals [100]. Furthermore, nausea and vomiting are more common with certain pathogens such as norovirus and shigella [101]. 

### 3.6. Psychological Stress and Anxiety

One potential cause of nausea in athletes that remains much understudied is the role of psychological stress and anxiety. Anecdotally, many elite athletes have dealt with bouts of pre-competition nausea with some examples including Bill Russell, Larry Bird, and Steve Young. A small number of observational studies have linked anxiety and/or stress to GI symptoms during exercise [102,103], but no published research has directly linked pre- or during-competition anxiety to the onset of nausea and vomiting. 

Still, evidence from other sources suggests that increases in pre-competition state anxiety would translate to more frequent or severe nausea in some athletes. Research done in a variety of clinical populations shows that anxiety and nausea are positively associated and that reducing stress and anxiety through relaxation interventions can lessen the severity of nausea [104,105,106,107,108]. Furthermore, among the general population, the presence of an anxiety disorder is associated with higher odds of experiencing nausea [109]. The links between stress, anxiety, and nausea are probably mediated through activation of the SNS and secretion of corticotropin-releasing factor from the hypothalamus, which reduces esophageal and gastric motility and slows gastric emptying [110].

### 3.7. Other Medical Issues

A variety of acute and chronic medical ailments can elicit nausea, and although the goal of this narrative review is not to serve as a comprehensive overview of all these conditions, some of them are described here for the reader’s reference. If an athlete complains of nausea during or after exercise, particularly when activity is conducted in a hot/humid environment, exertional heat illnesses should be considered as a potential underlying cause [111]. Other acute medical issues that are possible sources of nausea in athletes include myocardial infarction [112], severe pain and migraines [113], a head injury [113], appendicitis [113], and motion sickness [113], though there are many others. Chronic medical conditions are also potential causes of nausea, with some notable examples being gall bladder diseases, peptic ulcers, functional dyspepsia, and panic disorder [113,114]. Considering the nature of the athlete’s sport (e.g., football vs. distance running), contextual circumstances (e.g., environmental conditions), and the athlete’s personal medical history should help a practitioner identify which, if any, of these aforementioned medical issues is contributing to a given case of nausea. 

## 4. Prevention and Management of Nausea in Sport

Developing and implementing a plan to successfully prevent nausea will depend on the situation and athlete. Because there are so many potential causes of nausea, it’s important to identify the most likely culprits in a given situation. Table 1 provides an overview of prevention and mitigation strategies based on the suspected source of nausea. 

Beyond avoiding the triggers outlined in Table 1, another option for preventing or reducing the severity of nausea is to take antiemetic drugs. Ondansetron, which is an antiemetic from the 5-HT_3_ antagonist class of medications, is often kept on hand at endurance races for treating nausea and vomiting [115]. Anecdotally, ondansetron is effective for alleviating nausea in these situations, but to date, there is a lack of randomized trial data supporting its use as an antiemetic during exercise. 

## 5. Conclusions

Although nausea isn’t the most common GI symptom in the majority of sports, it is quite prevalent during short high-intensity exercise and ultra-endurance exercise. Furthermore, in comparison to more mild symptoms like belching and flatulence, nausea can impair competition performance. Known causes of nausea and vomiting during training and competition include catecholamine secretion, hypohydration, hyponatremia, altitude exposure, excessive fluid/food consumption, hypertonic beverage intake, pre-exercise consumption of fatty- or protein-rich foods, prolonged fasting, various supplements (caffeine, sodium bicarbonate, ketones), certain drugs (antibiotics, opioids), GI infections, and competition-related anxiety. Beyond directly addressing these sources, antiemetic drugs like ondansetron may also be useful for alleviating nausea, especially during ultra-endurance exercise. 

## Figures and Tables

**Table 1:** Causes of Nausea and Associated Prevention Strategies.

Sources of Nausea	Prevention/Mitigation Strategies
SNS activation and catecholamine secretion	Reduce exercise intensity Avoid high-dose caffeine and other stimulants Avoid prolonged fasting
Hypohydration and heat stress	If possible, drink enough fluid during exercise to prevent large body mass losses (i.e., >2–3% of mass) Acclimatize to thermally challenging environments Use pre- and per-cooling strategies to reduce the impact of heat stress
Altitude exposure	Pre-acclimatize to competition elevation Speak to a healthcare provider about drugs such as acetazolamide and metoclopramide
Excessive fluid intake	Avoid consuming large fluid volumes during exercise (≥750–1,000 mL/h)
Concentrated carbohydrate drinks	Consume only small amounts of hypertonic beverages during exercise Choose carbohydrate sources that contain a mix of glucose and fructose when consuming large amounts of carbohydrate (>50 g/h) during exercise
Inappropriate pre-exercise food choices	Limit fat and solid protein intake within 1–2 h of exercise Avoid large doses of fiber (>5–10 g) within 1 to 2 h of exercise
Supplements	If using caffeine, avoid large doses, particularly before stressful competition If using sodium bicarbonate, take 2–3 h before competition, co-ingest it with food, or use a multi-day regimen instead of an acute protocol If taking exogenous ketones, use an ester form Avoid very large dosages of sodium during exercise (>1–2 g/h) For other supplements that cause nausea (glycerol, medium chain triglycerides, etc.) reduce the amount, spread out doses, or take with food
Traveler’s diarrhea	Take precautions to reduce the chance of infection (avoid tap water, only eat food served piping hot, avoid raw foods, etc.) Speak to a healthcare provider about prophylactic antibiotic treatment
Competition stress and anxiety	Try relaxation techniques such as deep breathing and mindfulness meditation Consult with a sports psychologist
